# Genome-Wide Analysis and Molecular Characterization of Orf Virus Strain UPM/HSN-20 Isolated From Goat in Malaysia

**DOI:** 10.3389/fmicb.2022.877149

**Published:** 2022-07-11

**Authors:** Hassana Kyari Mangga, Jamilu Abubakar Bala, Krishnan Nair Balakrishnan, Alhaji Modu Bukar, Zaharaddeen Lawan, Auwal Gambo, Faez Firdaus Abdullah Jesse, Mustapha M. Noordin, Mohd-Lila Mohd-Azmi

**Affiliations:** ^1^Virology Unit, Department of Pathology and Microbiology, Faculty of Veterinary Medicine, Universiti Putra Malaysia, Serdang, Malaysia; ^2^Department of Veterinary Pathology and Microbiology, Faculty of Veterinary Medicine, Universiti Putra Malaysia, Serdang, Malaysia; ^3^Department of Microbiology, Faculty of Science, University of Maiduguri, Maiduguri, Nigeria; ^4^Department of Medical Laboratory Sciences, Faculty of Allied Health Sciences, Bayero University Kano, Kano, Nigeria; ^5^Department of Science Laboratory Technology, Ramat Polytechnic Maiduguri, Maiduguri, Nigeria; ^6^Department of Agricultural Technology, College of Agriculture, Hussaini Adamu Federal Polytechnic, Kazaure, Nigeria; ^7^Department of Microbiology, Faculty of Science, Usmanu Danfodiyo University Sokoto, Sokoto, Nigeria; ^8^Department of Veterinary Clinical Studies, Faculty of Veterinary Medicine, Universiti Putra Malaysia, Serdang, Malaysia

**Keywords:** ruminants, cell culture, genome, next-generation sequencing, Orf virus

## Abstract

Contagious ecthyma commonly known as Orf is a globally important, highly contagious zoonotic, transboundary disease that affects domestic and wild ruminants. The disease is of great economic significance causing an immense impact on animal health, welfare, productivity, and trade. Detailed analysis of the viral genome is crucial to further elucidate the molecular mechanism of Orf virus (ORFV) pathogenesis. In the present study, a confluent monolayer of lamb testicle cells was infected with the processed scab sample obtained from an infected goat. The presence of the virus was confirmed using polymerase chain reaction and electron microscopy, while its genome was sequenced using next-generation sequencing technology. The genome sequence of Malaysian ORFV strain UPM/HSN-20 was found to contain 132,124 bp with a G + C content of 63.7%. The homology analysis indicates that UPM/HSN-20 has a high level of identity 97.3–99.0% with the other reference ORFV strain. Phylogenetic analysis revealed that ORFV strain UPM/HSN-20 is genetically more closely related to ORFV strain XY and NP from China. The availability of the genome-wide analysis of ORFV UPM/HSN-20 strain from Malaysia will serve as a good platform for further understanding of genetic diversity, ORFV infection, and strategic development for control measures.

## Introduction

Orf virus (ORFV), Bovine papular stomatitis virus (BPSV), Pseudocowpox virus (PCPV), and Parapoxvirus of red deer in New Zealand (PVNZ), collectively known as parapoxviruses, are among the most economically significant causal agents of diseases in the livestock industry for decades (Hatcher et al., 2015; Bala et al., 2018a). ORFV is the causative agent of contagious ecthyma, or contagious pustular dermatitis virus was the first virus to be described in this group, the prototype of the genus Parapoxvirus, family Poxviridae. It is an ovoid, enveloped, double-stranded positive-sense DNA virus with a linear genome of 130–140 kb (Fleming et al., 2015; Sahu et al., 2020b). Its genome consists of two regions: the core and variable regions. The core region is conserved across most members of the poxvirus group and encodes essential proteins for structural components, nucleotide biosynthesis, genome replication, transcription, and assembly. On the other hand, the variable regions are found at the 5′ and 3′ terminal ends. The proteins encoded by these terminal genes are the major determinants of host range and viral pathogenesis (Delhon et al., 2004).

The disease has a worldwide distribution and mainly affects sheep and goats. But recently, cross-infection of other various domestic and wild ruminants has been reported in different parts of the world (Spyrou and Valiakos, 2015). The major route of transmission is *via* skin abrasion. After an incubation period of 8–14 days, the infected animals develop proliferative lesions commonly on the lips, muzzle, ears, eyelids, tongue, nostril, and occasionally the infection spreads to other non-wooly areas legs, feet, and udders (Chi et al., 2013). The disease is self-limiting, and lesions slowly heal within 1–2 months after progression from erythema, vesicle, pustules, papules, and scabs (Peralta et al., 2018). Epidemiological reports indicated morbidity is high, but mortality associated with CE is usually low. However, in severe cases, complications from fly infestation and secondary infection by bacteria and fungi have a significant impact leading to increased death of young animals, decrease in optimum productivity, and restriction of domestic and international trade (Bala et al., 2018b; Lawal et al., 2021). For zoonotic infection, in immunocompetent individuals, human Orf is localized and self-limiting, and disease resolves spontaneously within weeks (Hasheminasab et al., 2016; Kassa, 2021). In immunocompromised patients, complications such as lymphangitis, erythema multiforme, and toxic erythema have been reported (Lederman et al., 2007; Cubells et al., 2016).

In Malaysia, several studies have reported the occurrence of ORFV among sheep and goats from the diverse geographical region at a different time based on seroprevalence and molecular detection based on partial gene amplification (Al-Ajeeli, 1995; Abdullah et al., 2015; Sadiq et al., 2017; Bala et al., 2020). Recently, a high prevalence rate of 22.8% was reported during a serosurvey involving 504 animals in Terengganu (Bala et al., 2019). Another study reported an infection rate of 36.7 and 7.8% among goats and sheep from a small ruminant farm in Selangor (Jesse et al., 2018). To date, most of the published studies focused only on the partial sequence analysis based on conserved genes and seroprevalence studies. Despite the high incidence of the disease, there are no comprehensive molecular studies of the endemic ORFV affecting small ruminant farms in Malaysia. This is the first report on genome-wide sequence analysis of ORFV isolated in Malaysia and it is an effective approach to an enhanced understanding of the virus pathogenesis, epidemiology, and strategic development for control measures.

## Materials and Methods

### Sample Processing

Scab samples were collected from the tissue of an infected goat with clinical symptoms of Orf from a farm located in Selangor, Malaysia. The sample was processed according to the method described by Chi et al. (2015). One gram of scab was homogenized in 0.01 Phosphate-Buffered Saline (PBS). The suspension 10%w/v obtained was then centrifuged at 3000 rpm for 10 min. The supernatant was carefully aspirated and supplemented with penicillin (100 units/ml) and streptomycin (100 μg/ml) then stored at −80°C.

### Virus Propagation

Lamb testis cells obtained from ATCC (OA3.Ts CRL-6546) were maintained in Dulbecco’s Modified Eagle’s Medium (DMEM, GIBCO BRL Grand Island, United States), supplemented with 10% fetal bovine serum (FBS, GIBCO BRL Grand Island, United States), penicillin G (100 units/ml), and streptomycin (100 μg/ml). Confluent monolayer of the cells in 25 cm^3^ flask was infected with 100 μl of processed virus sample. The flask was placed in a CO_2_ incubator at 37C for 1 h. Fresh DMEM containing 1% FBS was added to the cells after washing with PBS. Frequently, the cells are observed daily for virus-induced cytopathic effect (CPE). When the CPE is 80%, the virus is harvested by methods described by Azmi and Field (1993) and Yang et al. (2014).

### Virus Concentration and Purification

After four consecutive passages of the ORFV isolate using a T25 cm^3^ flask, the virus was subsequently cultivated for purification. This is conducted by sucrose gradient ultracentrifugation as described by Guo et al. (2003). The viral supernatant obtained after three cycles of freezing and thawing was subjected to concentration using polyethylene glycol 6,000 (PEG 6000; Calbiochem, Darmstadt, Germany). We added 10 ml of 36% sucrose in TE buffer onto an ultracentrifuge tube, then slowly transferred the concentrated virus suspension and centrifuged at 65000 × g for 1 h at 4°C. The collected pellet was further purified using five different concentrations of sodium diatrizoate fractions at 50, 40, 30, 20, and 10% (w/v) in TE buffer. The white opalescent virus band detected was carefully aspirated from the gradient tubes and resuspended in PBS. The purified virus suspension was negatively stained with a drop of 2% phosphor-tungstic acid, followed by examination under a transmission electron microscope (TEM: Hitachi, H7100) at an accelerated voltage of 75 kV at microscopy unit at Institute of Bioscience, Universiti Putra Malaysia.

### DNA Extraction

Viral DNA from scab samples, cell cultures passages, and purified virions was extracted using a highly specific viral nucleic acid extraction kit (innuPREP virus DNA kit, Analytik Jena, Germany) according to the manufacturer’s instructions. The purity and concentration of the DNA were determined spectrophotometrically using BioPhotometer^™^ plus (Eppendorf, Hamburg Germany) at the ratio of 260 –280 nm.

### Polymerase Chain Reaction

A set of primer designated as forward, and reverse as previously described by Chi et al. (2013) was designed and synthesized by Integrated DNA Technology (forward: 5′-ATG TGG CCG TTC TCC TCT ATC-3′; Reverse: 5′-TTA ATT TAT TGG CTT GCA G-3′) for the amplification of fragments of major immunogenic envelope gene (B2L gene) of ORFV isolate. The polymerase chain reaction was carried out using Qiagen Kit (Qiagen, United States) following the manufacturer’s protocol. Briefly, PCR was performed containing 122 ng extracted DNA, 10 nmol of each primer, Taq Master Mix, and nuclease-free water. The thermal cycler was programmed based on the following cycling condition: the initial denaturation was at 94°C for 3 min, followed by the second cycle of denaturation at 94°C for 30 s; annealing temperature was 55°C for 30 s; extension at 72°C for 1 min; and the program was set to repeat from cycle 2 for 35 cycles and hold at 10°C. Gel electrophoresis of the PCR products was carried out using 1.25% agarose gel at 80 V for 35 min. The gel was stained with red safe, and DNA bands were viewed using a UV transilluminator (Gel Doc, BioRad).

### Genome Sequencing and Assembling

Viral DNA extracted from the purified virions was subjected to library preparation using Nextera DNA Flex Library Prep kit (Illumina Inc., United States) according to the manufacturer’s instruction. The libraries were sequenced using MiSeq technology (Illumina Inc., United States). Raw reads were filtered to remove artificial sequences and a Phred quality score of N30 was employed for quality control in BBDuk software (BBDuk version 36). MEGAHIT software (version 1.2.8) was used for *de novo* assembly of the paired-end reads to contigs. The contigs were further analyzed using RAST and Prodigal PROKKA software (version 1.14.0) for gene prediction.

### Open Reading Frame Analysis and Annotation

All the predicted proteins were then subjected to analysis using online Expasy software and BlastP at the National Center for Biotechnology Information^1^ to identify the gene family. Thus, the predicted ORFs are arranged, numbered, and named according to the reference ORFV strains. In addition, the percentage of nucleotide sequence among the isolates was determined using Clustal Omega (1). After annotation, to confirm the missing of ORF 001 and ORF 134 from the present isolate, specific primers were used for the amplification of a 928 bp region forward primer (GCCGTGGCCGAGTTGTAG) reverse primer (CTCGGTGACCTGCCTGAC) at the 5′ end and 1,172 bp region forward primer (TGCACCAGCATCTGCTAAAC) reverse primer (ACATTCCAAGCTCTCGTCGT) at the 3′ end of the genome.

### Phylogenetic Tree and Distance Matrix

An alignment containing 26 complete genome sequences of parapoxviruses and other closely related Squirrelpox virus and Molluscum Contagiosum virus as an outgroup based on the conserved gene (ORF111) was used for comparison. The Phylogenetic tree was constructed using the maximum likelihood methods and Tamura-Nei model (Tamura and Nei, 1993). The reliability of the tree was assessed by a bootstrap test with 1,000 replicates, constructed with MEGA-X (Kumar et al., 2015). The identity value of the nucleotide sequence among parapoxviruses and other selected poxviruses was calculated using the pairwise distance method using MEGA-X software.

### Genetic Diversity and the Host

Another phylogenetic tree was constructed using a gene at the variable region (ORF008) to determine the relationship of the ORFVs included in this study base on host. The Phylogenetic tree was constructed using the maximum likelihood methods and Tamura-Nei model (Tamura and Nei, 1993). The reliability of the tree was assessed by a bootstrap test with 1,000 replicates, constructed with MEGA-X (Kumar et al., 2015). The sequences of the ORF117 encoding granulocyte-macrophage colony-stimulating factor (GM-CSF) inhibitory factor were analyzed to study virus genetic diversity in relation to host using Bioedit bioinformatic software.

## Results

### Virus Isolation and Identification

**Figure 1:**
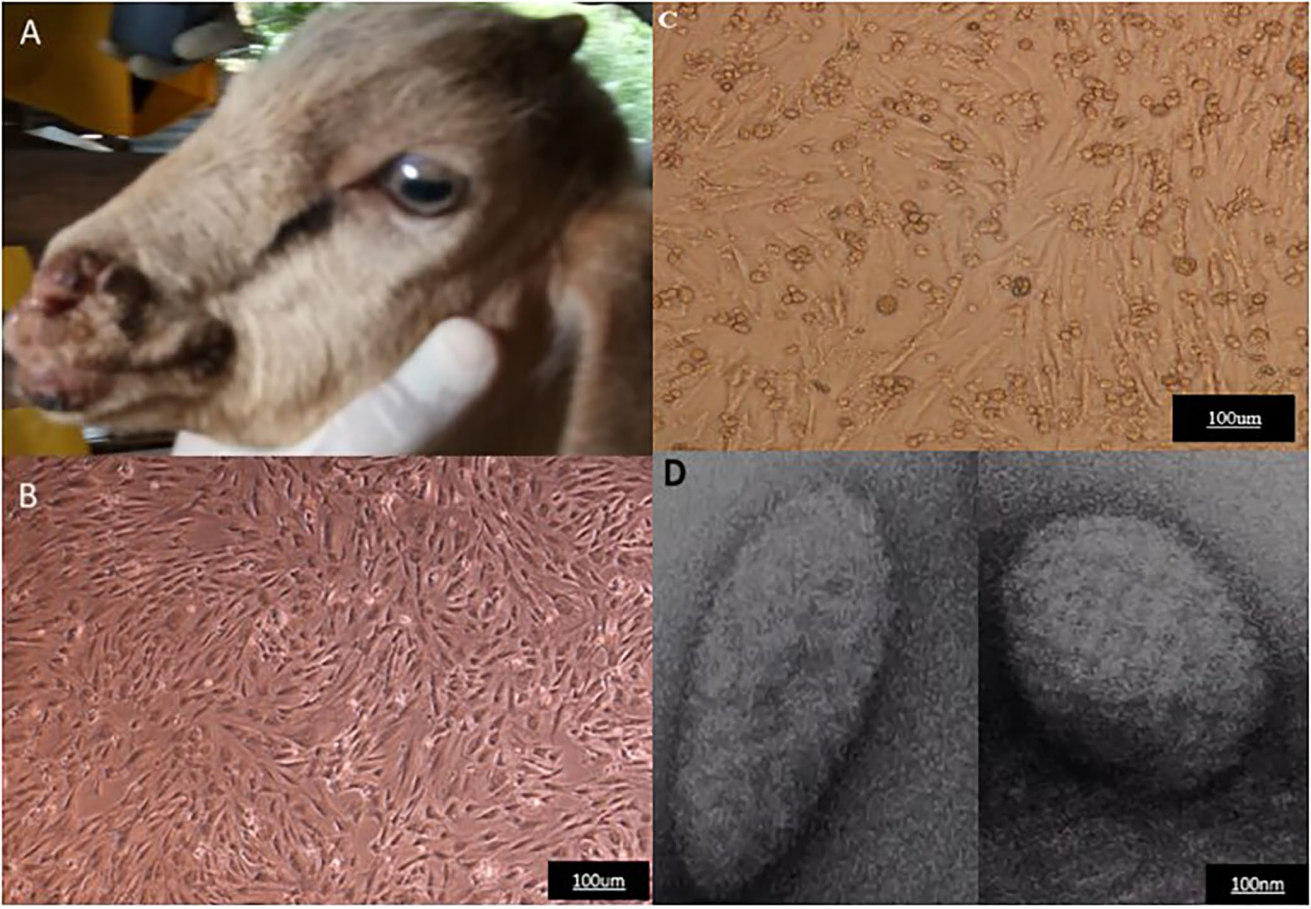
Representative of clinical case of Orf virus (ORFV) infection, Cytopathic effect following ORFV infection of LT cells, and electron microscopy of the purified virus. **(A)** Goat showing multiple lesions. **(B)** Uninoculated LT cells. **(C)** Infected cells with cytopathic effect post-inoculation. **(D)** Electron micrograph of the purified virus.

The clinical sample collected from a goat suspected of contagious ecthyma infection was processed using standard procedure. A confluent monolayer of LT cells infected with the processed sample displays typical characteristics of ORFV CPE. These morphological changes include ballooning, rounding, and degeneration of cells which begins to appear 3 days post-inoculation. The CPE becomes more pronounced in 6–7 days with an increase in cytoplasmic granulation and 70–80% cell detachment. Purification of the concentrated virus using sodium diatrizoate density gradient (Robinson et al., 1982; Guo et al., 2003) revealed a distinct white opalescent band typical of ORFV appeared between 40 and 30% at approximately 36%. Total DNA extracted from the scab samples, cell culture passages, and purified virions particles were subjected to molecular analysis. PCR amplification using a specific primer targeting the B2L gene produces positive bands at the expected region of approximately 1,137 bp from all the samples. Transmission electron microscopic examination of the purified virion reveals the presence of negatively stained virion particles (Figure 1).

### Genome Sequence Analysis

Orf virus strain UPM/HSN-20 was successfully sequenced in this study using next-generation sequencing (NGS) on the Illumina platform. The raw reads were processed and mapped to the respective reference genome. *De novo* assembly of the quality sequence reads revealed a linear contiguous sequence of 132,124 bp with G + C content of 63.7% (Table 1). The assembly demonstrates high levels of identity 97.3–99.0% with the other reference ORFV strains deposited in the GenBank. Whereas, the percentage’s identity with other members of the parapoxviruses ranges from 78.2 to 93.8% when compared using NCBI BLAST. The open reading frames (ORFs) were predicted using RAST and BLASTP. A total of 127 predicted protein best hits with ORFV reference strain; the nucleotide sequence of ORF 002, 005, and 132 has low hit, while two genes 001 at the 5′ end and 134 at 3′ variable ends were confirmed to be deleted using comparative sequence analysis and absence of amplification. The order and numbering of the predicted genes including the newly recognized genes (12.5 and 107.5) were adopted from Mercer et al. (2006).

**Table 1:** List of reference parapoxviruses used in this study.

NO	Virus	Strain	Country of isolation	Animal	Predicted genes	Average G + C	Genome size (kbp)	GenBank accession no.	References
1	ORFV	HSN-20	Malaysia	Goat	127	63.7	132,124	MW537048	This study
2	ORFV	SAOO	United States	Kids	130	63.4	139,962	AY386264	Delhon et al., 2004
3	ORFV	IA82	United States	Lamb	130	64.3	137,241	AY386263	Delhon et al., 2004
4	ORFV	NZ2	New Zealand	Sheep	132	64.0	137,820	DQ184476	Mercer et al., 2006
5	ORFV	D1701	Germany	Sheep	288	63.7	134,038	HM133903	McGuire et al., 2012
6	ORFV	XY	China	Goat	132	63.8	138,321	KP010353	Chi et al., 2015
7	ORFV	GO	China	Goat	132	63.6	139,866	KP010354	Chi et al., 2015
8	ORFV	NP	China	Goat	124	63.8	132,111	KP010355	Chi et al., 2015
9	ORFV	SJ1	China	Goat	129	63.6	139,112	KP010356	Chi et al., 2015
10	ORFV	NA1/11	China	Lamb	132	63.6	137,080	KF234407	Li et al., 2015
11	ORFV	HN3/12	China	Sheep	132	63.7	136,643	KY053526	Chen et al., 2017
12	ORFV	SY17	China	Sheep	131	63.8	140,413	MG712417	Zhong et al., 2019
13	ORFV	NA17	China	Goat	132	63.7	139,287	MG674916	Zhong et al., 2019
14	ORFV	GZ18	China	Goat	130	63.9	137,986	MN648218	Unpublished
15	ORFV	CL18	China	Sheep	129	63.8	138,495	MN648219	Unpublished
16	ORFV	MP	India	Goat	132	63.7	139,807	MT332357	Sahu et al., 2020a
17	ORFV	TVL	United States	Sheep	-	64.1	134,893	MN454854	Heare et al., 2020
18	ORFV	IHUMI-1	France	Human	126	64.1	132,823	LR594616	Andreani et al., 2019
19	PCPV	VR634	Finland	Human	134	64.4	145,289	GQ329670	Hautaniemi et al., 2010
20	PCPV	F00.120R	Finland	Reindeer	131	65.0	133,169	GQ329669	Hautaniemi et al., 2010
21	BPSV	TX09c1	United States	Cow	129	64.1	135,072	KM875472	Huang et al., 2015
22	BPSV	ARO2	United States	Calf	133	64.4	134,431	AY386265	Delhon et al., 2004
23	PVNZ	HL953	Germany	Red deer	131	64.5	139,981	KM502564	Friederichs et al., 2015
24	Seal Pox	AFK76s1	Poland	Gray seal	119	55.9	127,941	KY382358	Günther et al., 2017
25	SPV	SPRS	UK	Squirrel	139	66.0	148,803	HE601899	McInnes et al., 2006
26	MCV	MCV2	Slovenia	Human	170	–	189,257	MH320556	Zorec et al., 2018

**Figure 2:**
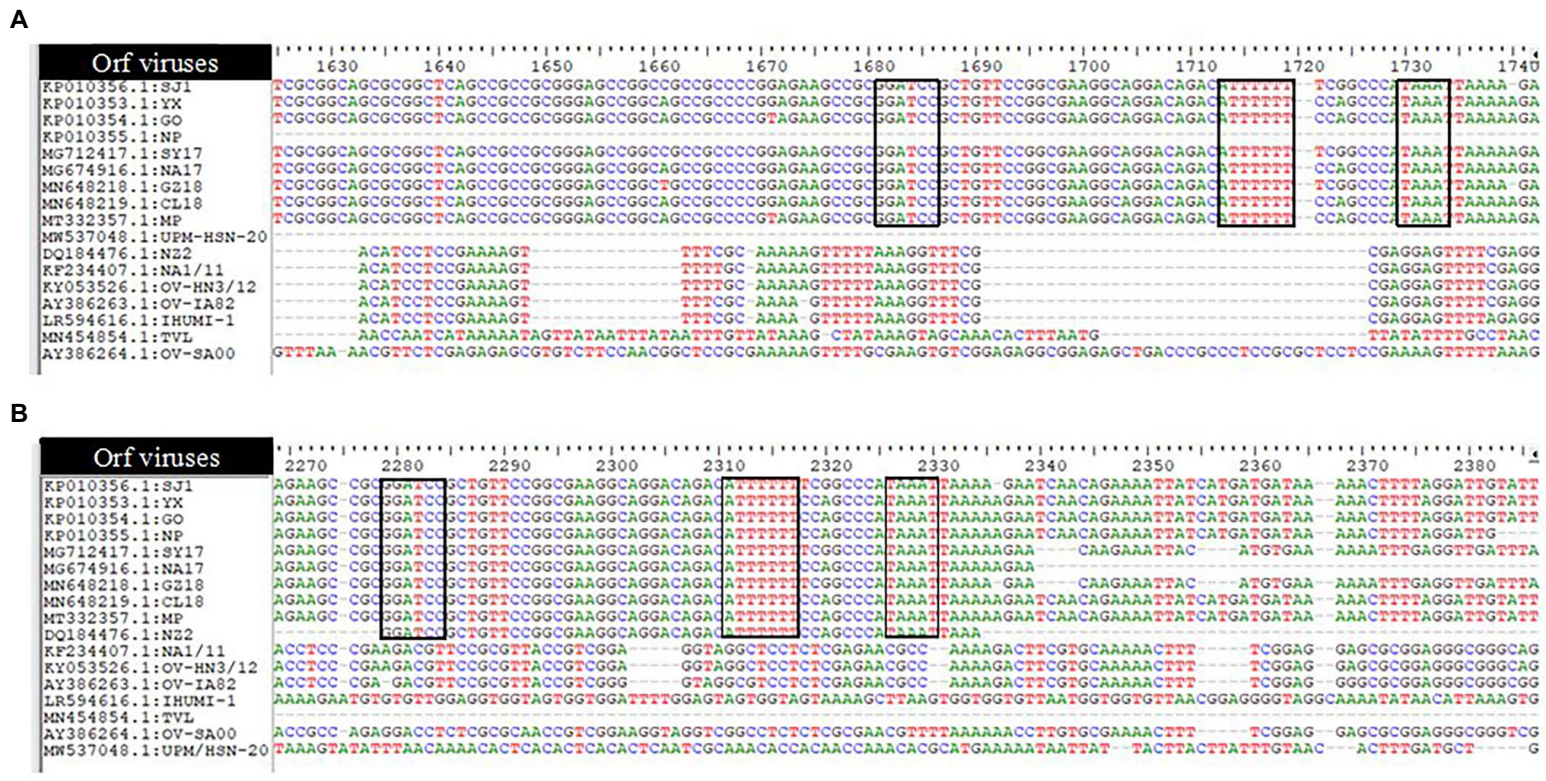
Analysis of ITRs of 17 Orfvimses **(A)** the left end (5') sequence alignment **(B)** the right end (3') sequence alignment. The tenninal BamHI site, telomere resolution motif are indicated with black box (ATTTTTT-N(8)-TAAAT).

Like other poxviruses, the terminal regions of the ORFV genome are bounded by inverted terminal repeats (ITR). The analysis of ITR sequences of 17 ORFV strains revealed sequence variability (Figure 2). The left end (5′) sequence of SJ1, YX, GO, SY17, NA17, GZ18, CL18, and MP contained terminal BamHI site along with telomere resolution motifs (ATTTTTT-N(8)-TAAAT), as indicated with the black box (A), while NP, NZ2, NA1/11, OV-HN3/12, OV-IA82 and IHUMI-1, UPM/HSN-20, TVL, and OV-SA00 lack this motif. The right end (3′) sequence of SJ1, YX, GO, NP, SY17, NA17, GZ18, CL18, MP, and NZ2 contained terminal BamHI site along with telomere resolution motifs (ATTTTTT-N(8)-TAAAT), as indicated with the black box (B), while NA1/11, OV-HN3/12, OV-IA82 and IHUMI-1, UPM/HSN-20, TVL, and OV-SA00 lack this motif.

### Phylogenetic and Distance Analysis

**Figure 3:**
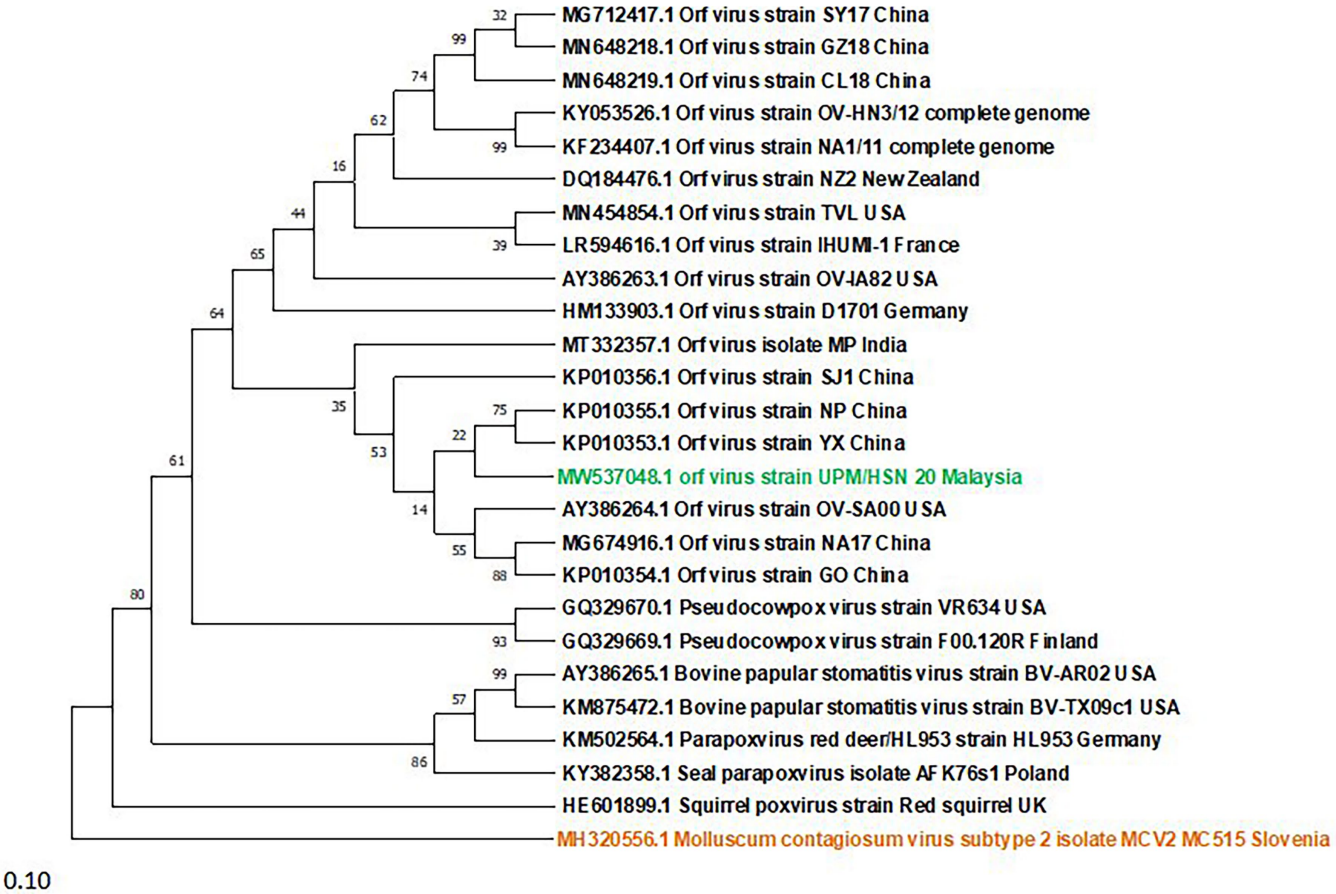
Phylogenetic tree based on highly conserved ORFIII gene of parapoxviruses and other closely related poxviruses. The tree was constructed using the maximum likelihood methods and Tamura-Nei model with 1000 bootstrap replicates in NIEGA-X software.

A Phylogenetic tree was constructed for tracing the evolutionary relationship between parapoxviruses and closely related poxviruses using highly conserved chordopoxvirus conserved ORF111 gene. The most closely related strain with isolates in this present study based on the highly conserved gene is XY and NP ORFV strain from China, as shown in Figure 3. The phylogenetic tree also revealed that ORFVs are distantly related to Squirrelpox virus, while the Molluscum Contagiosum virus appears as an outgroup. In the case of the pairwise analysis based on ORF111, the result shows a lesser mutation rate among ORFVs, while the other parapoxviruses has shown greater genetic variation Table 2.

**Table 2:** Estimates of Evolutionary Divergence between Orf virus UPM/HSN-20 and other reference isolates.

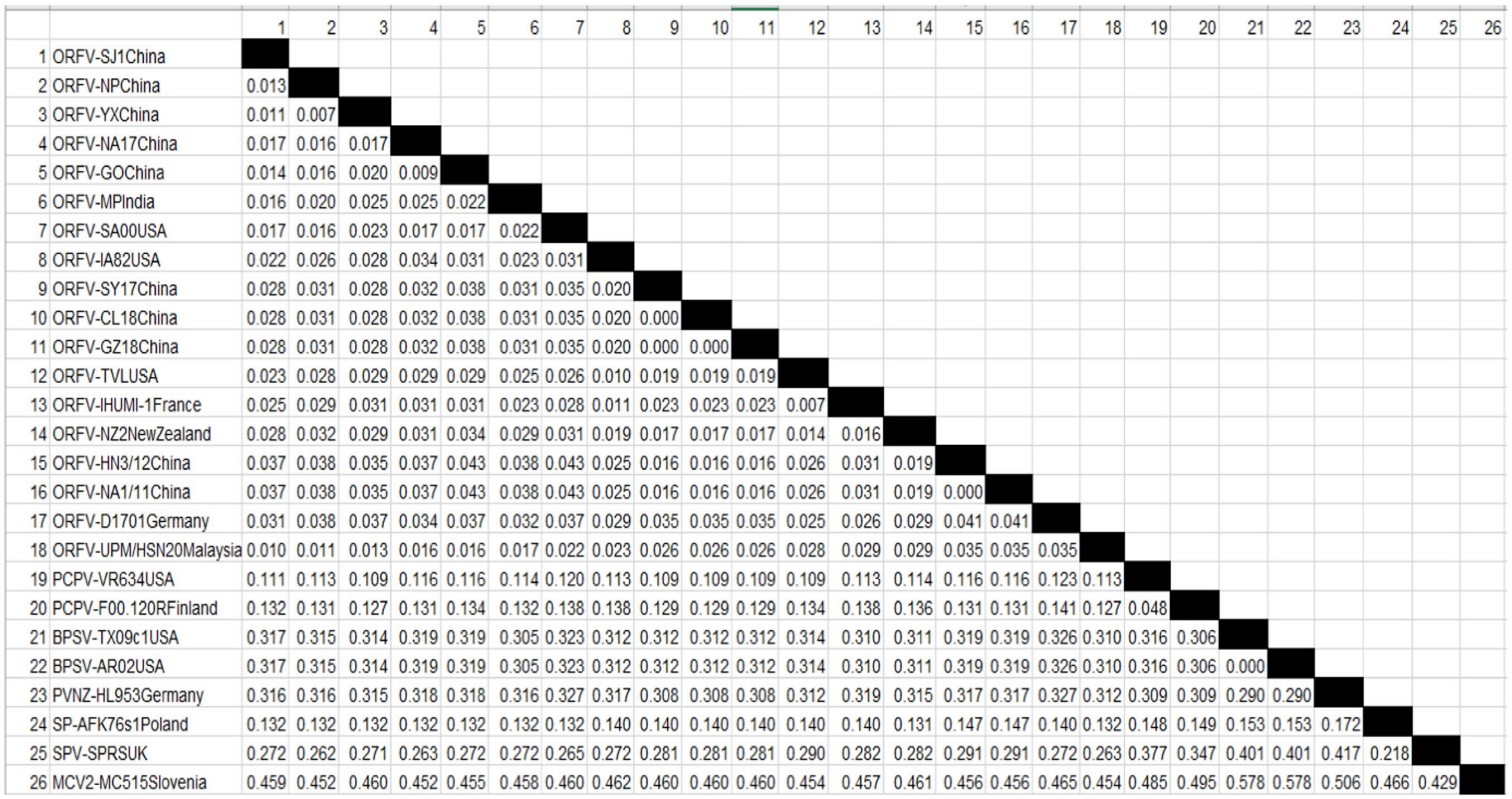

### Genetic Diversity and Host

**Figure 4:**
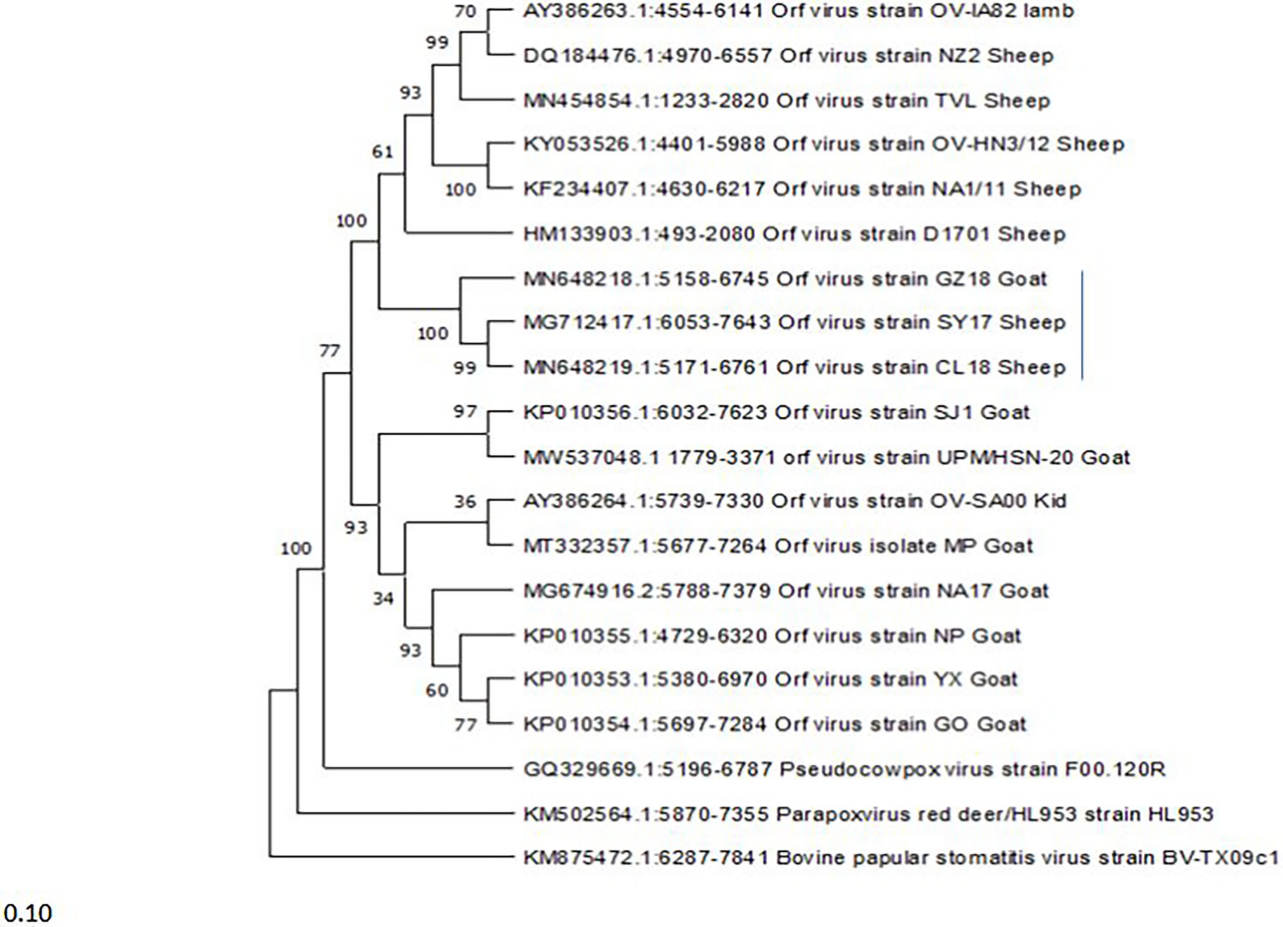
Phylogenetic tree based on ORF008 of parapoxviruses. The tree was constructed using the maximum likelihood methods and Tamura-Nei model with 1000 bootstrap replicates in NIEGA-X software.

A phylogenetic tree was constructed to investigate the relationship between viruses and the infected host based on the ORF008 gene. The ORFVs form two major clusters (Figure 4), with the lower major cluster, consisting of eight (8) isolates all originating from goats (SJ1, UPM/HSN-20, OV-SA00, MP, NA17, NP, XY, and GO), while the upper major cluster consists of nine (9) ORFVs, eight (8) isolates originating from sheep (OV-IA82, NZ2, TVL, OV-HN3/12, NA1/11, D1701, SY17, and CL18) and one (1) isolate originating from goat (GZ18). The GZ18 strain forms a sub-cluster with SY17 and CL18 with strains originating from sheep.

**Figure 5:**
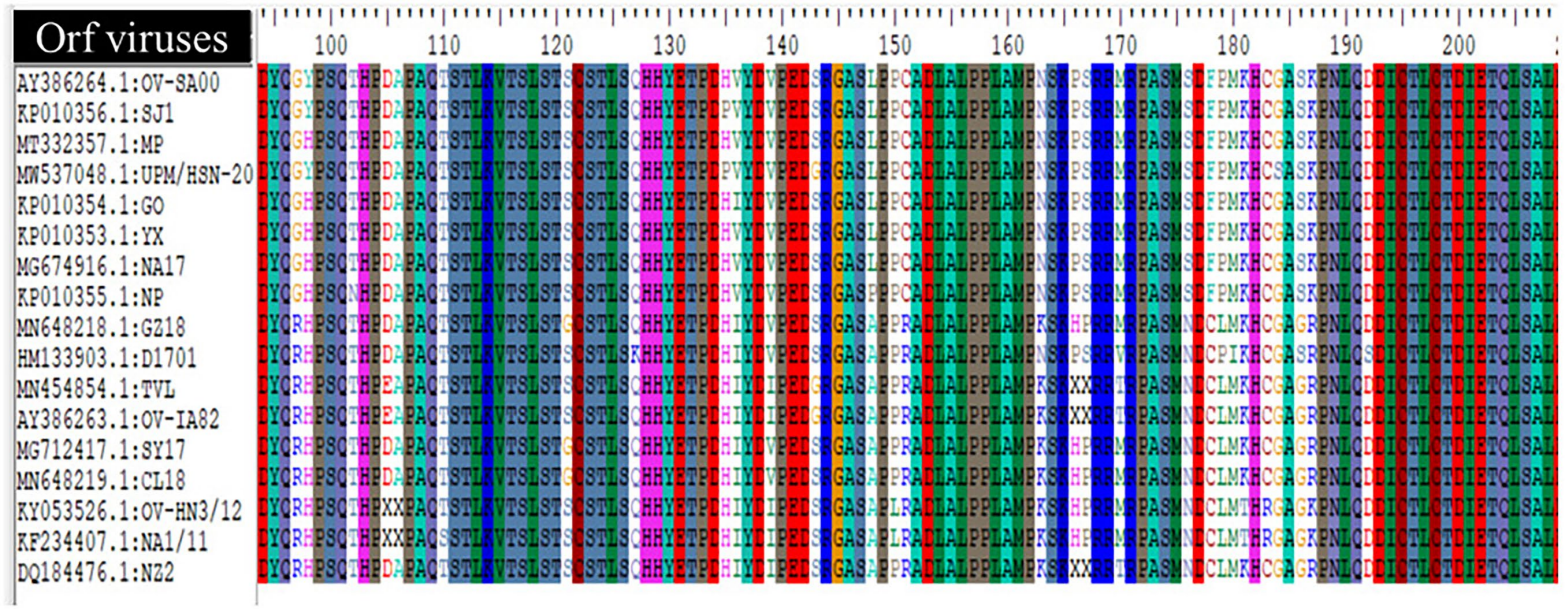
Multiple alignment of deduced amino acid sequence (ORF117) of Orf virus UPM/HSN-20 and other reference isolated.

The comparison of amino acids sequences of the ORF117 gene among 17 ORFV strains highlighted that the following strains such as GZ18, D1701, TVL, OV-IA82, SY17, CL18, OV-HN3/12, NA1/11, and NZ2 contained Arginine at position 97, Arginine at position 151, Asparagine at position 176, and Cysteine at position 178, while other strains consist Glycine, Cysteine, Serine, and Phenylalanine in that position (Figure 5). In addition, Alanine was found at position 148 in GZ18, D1701, TVL, OV-IA82, SY17, CL18, OV-HN3/12, NA1/11, and NZ2, while other strains contained Leucine except NP presented Proline.

## Discussion

Livestock farming plays an important role in the development of the agricultural economy and is essential to the livelihoods of millions of farmers in developing countries (Dror and Allen, 2011; Wodajo et al., 2020). It is a well-known fact that the progress of this agricultural sub-sector is mainly determined by the health status of the animal which, in turn, depends upon the intake of high-quality feed and prevention of occurrence of infectious diseases among herds. In Malaysia, ORFV has been recognized for many years as an important pathogen affecting the growth of the livestock industry (Al-Ajeeli, 1995; Bala et al., 2019, 2020). To limit the impact of the outbreaks and to take efficient steps toward control and eradication of the disease, studies on molecular heterogeneity of ORFVs circulation are of utmost importance.

The infected goat is presented with pyrexia and coalescing papillary, severe ulcerative, and scabby lesions of various sizes on the epidermis of the muzzle and lips (Figure 1). During the outbreak, two animals were severely affected, one died, while the other survived under specialized veterinary care. Other clinical symptoms observed include nasal discharge, painful cough, difficulty in breathing, and slow movement. Previous clinical case studies described that infected Boar goats developed multifocal, severe proliferative dermatitis accompanied by other clinical conditions including chronic pneumonia, arthritis, and lymphadenopathy (Nandi et al., 2011; Sadiq et al., 2017). The clinical signs and symptoms observed in this study are consistent with the characteristics of ORFV infection.

To support the clinical diagnosis, a confluent monolayer of LT cells was infected with the processed sample. Virus propagation and isolation in cell culture are regarded as a “gold standard” for the detection of poxviruses (Grossegesse et al., 2017). Although the method is technical, time-consuming, and does not provide rapid diagnosis of infectious diseases, it is the only reliable method for generating sufficient stock of viruses for further studies. Various primary cells and continuous cell lines including lamb testis cell, Madin-Darby bovine kidney (MDBK), and Madin-Darby ovine kidney (MDOK) have been reported to be suitable for the cultivation of ORFVs (Abdullah et al., 2015; Kumar et al., 2018; Wang G. et al., 2019). Viral replication is followed by morphological and biochemical changes which ultimately lead to cell destruction (Zamri-Saad et al., 1999; Bala et al., 2020). In this study, typical characteristics of ORFV growth in cell culture were observed in LT cells line similar to earlier reports (Hematian et al., 2016; Lawal et al., 2021). These morphological changes commonly induced by ORFV include rounding and enlargement of cells together with swelling known as ballooning which indicates the starting point of CPE and begins to appear as early as 3 days post-inoculation. The continuity of this process leads to advanced CPE where the accumulated swollen and enlarged cells clumps and detaches from the flask.

Cytopathic effect alone is not sufficient for identification of the presence of the virus because several factors can alter the results, an additional test is needed for visualization of virus particles. The introduction of negative staining and the wider availability of electron microscopes have made electron microscopy essential in the differential diagnostic cell culture and clinical samples (Hazelton and Gelderblom, 2003; Shen et al., 2013). Pattern recognition of the size and particle morphology leads to direct and rapid visualization of an infectious agent (Gelderblom and Männel, 2003; Dal Pozzo et al., 2011). Some recent studies have employed the use of transmission electron microscopy for *in vivo* and *in vitro* identification of ORFV from clinical suspension by negative staining (Nashiruddullah et al., 2018; Galante et al., 2019). Therefore, this study has utilized this technique to ascertain the presence of the virus following the purification procedure. Large virion particles with typical morphological characteristics of parapoxviruses were observed (Figure 1). The enveloped virions presented as an ovoid shape with a unique spiral crisscross pattern that clearly distinguishes parapoxvirus genera from other poxvirus genera. For example, the TEM method has been useful for differential diagnosis between ORFV and capripoxviruses, all of which cause disease with similar clinical manifestation in sheep and goats (Rajesh and Swathi, 2018).

Next, to elucidate the genomic features of ORFV strain UPM/HSN-20, a high-throughput DNA-sequencing approach was employed, a technique that proved to be efficient in revealing detailed genetic information of an organism. In addition to identifying pathogens faster, NGS technology and bioinformatics can provide new insights into the molecular mechanism of microbial pathogenesis (Balakrishnan et al., 2017; Gwinn et al., 2019). Compared to Sanger sequencing, NGS technology has enabled researchers to study and understand the microbial world from broader and deeper perspectives. Pathogen’s genomics and epidemiology enhance public health efforts in preventing transmission of infectious diseases (Mohd-Azmi et al., 2000; Cao et al., 2017). Sequence analysis of our isolate revealed a linear sequence of 132,124 bp (GenBank accession number MW537048) with G + C content is 63.7%. All these features are consistent with the reference strain. The genome appears to be typical of PPVs, containing a central conserved region and variable region with a genus-specific GC profile (Chi et al., 2015; Zhong et al., 2019). Comparative sequence analysis of ORFV strain UPM/HSN-20 with the reference data available reveals a high degree of identity 97.3–99.0% among ORFVs. In addition, our sequence analysis indicates that PCPVs are genetically more closely related to ORFV in comparison to BPSV. Even though, BPSV and PCPV share the same host, whereas ORFV natural hosts are sheep and goats (Hautaniemi et al., 2010). The NCBI blast analysis revealed that the average nucleotide sequence identity between ORFV and PCPV is approximately 93% in contrast to 85% identity between ORFV and BPSV.

We discovered the deletion of two genes in the genome’s terminal regions in the present study (001 at the left end and 134 at the right end). Chi et al. (2015) previously reported similar deletion in the ORFV strain’s genome terminal region (NP: KP010355). The core part of the genome is well conserved, the terminal regions show significant variability and surprising evidence of rearrangement of the terminal sequences. Monkeypox virus was the first poxvirus to show genome rearrangement between non-homologous sequences (Esposito et al., 1981). Our knowledge of genome rearrangement resulting in duplication, transposition, and deletion is still in its infancy, and relatively few studies have reported sequence deletion among less and highly adapted cell culture ORFV isolates (Fleming et al., 1995; Cottone et al., 1998; McInnes et al., 2001; Chi et al., 2015).

To determine the evolutionary pathways and connections between ORFVs and other groups of closely related poxviruses, a phylogenetic tree was constructed. A molecular phylogenetic tree is a functional tool scientists use to show the evolutionary history of a set of species or groups of organisms (McLennan, 2010). From the literature as well as looking at the genome map, we found that ORFV ORF111 gene, formerly known as A35 gene (Rehm and Roper, 2011) by the vaccinia virus designation, is highly conserved in all mammalian-tropic poxvirus and has no similarity to non-poxvirus proteins, giving no clues as to its function. However, the bioinformatics analysis we conducted reveals that the ORFV ORF111 protein is antigenic based on VaxiJen protein function prediction software. In addition, the wide conservation of the ORF111 gene among all poxviruses with a mammalian host range suggests that this protein has an important role. Since the ORF111 gene is highly conserved, we constructed a phylogenetic tree to understand the relationship between parapoxviruses and other closely related poxviruses having high GC content. The phylogenetic tree constructed (Figure 2) in this study showed ORFV strain formed two separate branches on the tree, with the isolate under study (MW537048) forming a distinct cluster with XY (KP010353) and NP (KP010355) with Chinese isolates, signifying a common ancestor. Indeed, the Malaysian isolate (MW537048) and the two Chinese isolates (KP010353 and KP010355) are so genetically related that they are believed to share a common ancestor during evolution. The phylogenetic tree also revealed that ORFVs are distantly related to Squirrelpox virus, while the Molluscum Contagiosum virus appears as an outgroup. This is consistent with previous reports on evolutionary analysis of ORFVs and other poxviruses (Andreani et al., 2019). Besides the phylogenetic tree, the pairwise distance is one of the most widely used methods for the determination of mutation rates among isolates and/or groups of organisms. As indicated in Table 2, a lesser mutation rate was observed among ORFVs. The isolate in this study shows a closer identity with Chinese isolates. In addition, generally, higher genetic variation is observed between Orf viruses and other parapoxviruses. Mutations are a mandatory event for survival as such the local environment and host are important determining factors for virus growth and survival.

To further understand ORFV genetic diversity based on host predilection, a phylogenetic tree was constructed using the ORF008 gene. The ORF008 gene was among the genes used in previous studies conducted by Chi et al. (2015), and Zhong et al. (2019), which show ORFV strain diversity in relation to originating host. The tree they constructed in their studies shows that Orf viruses that were isolated from Sheep cluster together and those isolated from goats cluster together (Chi et al., 2015; Velazquez-Salinas et al., 2018; Zhong et al., 2019), suggesting that different genes in the genome of ORFV may be associated with the host predilection. While in the present study, we found that one goat isolate forms a cluster with two sheep isolates (GZ18, SY17, and CL18). These three isolates share a significant sequence similarity of 99.5%. However, considering the limited ORFV genome sequenced data available worldwide in the database, more studies are needed to confirm the virus diversity in relation to the host. In addition, ORFV infects not only sheep and goats (Spyrou and Valiakos, 2015), and including other hosts will greatly enhance the understanding of virus genetic diversity in relation to the host.

The genes in the conserved region perform essential functions and some genes such as B2L and F1L encoded by ORF11 and ORF059 have been widely used for rapid clinical diagnosis of ORFV (Yogisharadhya et al., 2017, 2018). While the variable region encodes some proteins that interfere with the early pathogen recognition, antiviral response, host’s cytokines, and cellular signaling, leading to pro-inflammatory responses to promote viral survival and replication. An example of such protein is a granulocyte-macrophage colony-stimulating factor (GM-CSF) encoded by the ORF117 gene. The GM-CSF inhibitory factor inhibits the downstream antiviral signaling of granulocyte-macrophage colony-stimulating factor and interleukin-2 (Wang R. et al., 2019). Interestingly, the amino acid sequence alignment of this biological significance protein reveals some unique amino acid sequence variations among ORFV strains (Figure 5). The effect of this sequence variation among the ORFV strain is yet to be reported. It is a well-known fact that amino acid changes can lead to changes in protein structure or function, which can sometimes affect the pathogenic characteristics of the virus.

## Conclusion

The present study has demonstrated the functional merit of both non-molecular and molecular approaches for the identification of ORFV obtained from a clinical specimen. Our study provided an overall genomic mapping of the recent Malaysian ORFV strain UPM-HSN-20. A comprehensive study has found that the putative biological characteristics of the Malaysian isolates were similar to those of other Orf viruses. Multiple sequence and phylogenetic analyses revealed that our isolate in this study is closely related to two ORFV isolates NP and XY from China. The analysis of ITR sequences of 17 ORFV strains revealed significant sequence variability. The study has provided useful information for further comprehensive understanding of ORFV molecular biology, epidemiology, and control of the disease.

## Data Availability Statement

The datasets presented in this study can be found in online repositories. The names of the repository/repositories and accession number(s) can be found at: https://www.ncbi.nlm.nih.gov/, MW537048.

## Author Contributions

M-LM-A, HM, JB, and KB participated in conceptualization of the idea and study design. HM, JB, and KB performed the experiments. HM, AB, ZL, and AG participated in the data analysis, interpretation, and preparation of manuscript draft. FJ and MN supervised the analysis and edited the manuscript. All authors contributed to the article and approved the submitted version.

## Funding

This work was supported by grants from Malaysian Skim Geran Penyelidikan Fundamental (FRGS) FASA1/2019 Universiti Putra Malaysia: FRGS/1/2019/STG03/UPM/01/2.

## Conflict of Interest

The authors declare that the research was conducted in the absence of any commercial or financial relationships that could be construed as a potential conflict of interest.

## Publisher’s Note

All claims expressed in this article are solely those of the authors and do not necessarily represent those of their affiliated organizations, or those of the publisher, the editors and the reviewers. Any product that may be evaluated in this article, or claim that may be made by its manufacturer, is not guaranteed or endorsed by the publisher.

## Footnotes

^1^ https://blast.ncbi.nlm.nih.gov/
